# Implementation of Flexibilities to the National School Lunch and Breakfast Programs and Their Impact on Schools in Missouri

**DOI:** 10.3390/nu15030720

**Published:** 2023-01-31

**Authors:** Sarah Moreland Russell, Jason Jabbari, Louise Farah Saliba, Dan Ferris, Eliot Jost, Tyler Frank, Yung Chun

**Affiliations:** 1 Prevention Research Center, Brown School, Washington University in St. Louis, One Brookings Drive, St. Louis, MO 63130, USA; 2 Social Policy Institute, Brown School, Washington University in St. Louis, One Brookings Drive, St. Louis, MO 63130, USA

**Keywords:** US federal nutrition policy, waivers, flexibilities, implementation

## Abstract

Background: In 2018, the United States Department of Agriculture (USDA) issued flexibilities to the National School Lunch and Breakfast Programs, relaxing the nutrition standards for milk, whole grains, and sodium. This study examines the implementation decision-making among Missouri school food services and the impact of implementing these flexibilities on the meals served. Methods: We developed a survey using the Consolidated Framework of Implementation to determine schools’ implementation of the flexibilities and factors related to implementation. To determine how the implementation of flexibilities affected participation, we merged the survey results with school-level meal county data from the Missouri Department of Elementary and Secondary Education. We used ordinary least squares regression to examine how flexibility adoption related to the number of meals served. Results: Most schools implemented the wheat, milk, and sodium flexibilities. Common reasons for implementation were increasing participation, meeting students’ preferences, expanding menu variety, and saving money. The implementation of flexibilities was associated with more lunches and breakfasts being served per month, particularly among free and reduced-price meals. Conclusions: Continued research is needed to determine how the increased uptake of school meals that do not fully meet dietary guidelines by low-income students results in inequities in health outcomes. The findings can inform the design and implementation of future policies, especially as new rules related to flexibility design are determined.

## 1. Introduction

The prevalence of childhood and youth obesity and overweight in the U.S. has nearly doubled in the past 20 years [1,2,3]. Obesity contributes to adverse health conditions, which increase morbidity, reduce quality of life, and result in significant short and long-term healthcare-related costs [4,5,6,7,8,9]. Among the health conditions linked with being overweight in school-aged children, are asthma, joint problems, type 2 diabetes, depression and anxiety, and sleep apnea [10,11,12,13,14]. Children who suffer from obesity and being overweight can experience impaired school performance in many ways, including higher rates of health-related absenteeism and social problems, such as low self-esteem [10,11,12,15,16,17,18,19,20]. Unfortunately, most youth in the U.S. do not meet the recommended servings of foods that provide proper nutrition, optimal growth, and development (i.e., fruits, vegetables, and whole grains) and consume too many of the foods that lead to poor health outcomes (i.e., foods high in sodium, saturated fat, and sugar) [21,22]. Poor nutrition also disparately affects minority children and children living in under-resourced households, putting these populations at the greatest risk for obesity and diet-related disparities [23].

School meal programs, such as the National School Lunch Program (NSLP) and School Breakfast Program (SBP), have been found to reduce food insecurity while improving nutrient intake, health, and education outcomes. These programs have significant reach, with an estimated 30 million students served lunch and 15 million served breakfast daily. When considering that school meals are a significant source of nutrition for children and that the implementation of various food policies can impact the nutritional quality of food, it is critical to understand how school meal policies might relate to student nutrition. Both the nutritional quality of food served in schools, as well as the number of meals being served, can have important implications for either narrowing or widening disparities in child health [23].

In an effort to combat childhood obesity, the Healthy Hunger-Free Kids Act (HHFKA-2010) was passed with bipartisan support in 2010, resulting in the final rule, Nutrition Standards in the National School Lunch and Breakfast Programs (NSLP/SBP) [24]. Prior to HHFKA, the mean sodium content of school lunches served was nearly twice the level recommended in the 2005 Dietary Guidelines, and saturated fat standards were only being met by 30% of schools [25]. The HHFKA updated school meal requirements and aligned the NSLP/SBP with nutritional science and evidence-based standards [26].

The new HHFKA regulations came into effect in the 2012/2013 school year [27,28,29,30,31]. The improvements aimed to increase the availability of fruits, vegetables, whole grains, and fat-free milk; reduce the levels of sodium and saturated and trans fat; and meet the nutrition needs of school children within calorie requirements [24]. The preliminary research results demonstrated the effectiveness of this large-scale public health policy, and studies utilizing School Nutrition and Meal Cost Study data from 2014–2015 found significant improvement in the nutritional quality of school meals [29,30,32]. Although implementing such broad-scale changes faced initial challenges, research shows that schools implemented the updated nutrition standards and began to offer healthier meals with significant reductions in sodium and the percentage of calories from saturated fat, as well as increases in fiber [31,33,34,35,36]. The HHFKA implementation was associated with a decline in fat and added sugar intake, as well as the prevalence of obesity and overweight for students who ate school lunches [37]. Additionally, one study using a nationally representative sample found boys eating school lunches post-HHFKA had lower body mass index (BMI) growth than nonparticipants [38]. Further research found that under the new guidelines, students liked the meals and ate more of them [39,40]. In a more recent study, Kenney et al. [29] tested whether the HHFKA was associated with reductions in child obesity risk over time. While the authors found no significant association between the legislation and childhood obesity trends overall, for children in poverty, the risk of obesity declined substantially each year after the act’s implementation [29]. The authors also report that obesity prevalence would have been 47 percent higher in 2018 if the HHFKA had not been implemented. These results suggest that the HHFKA science-based nutritional standards should be maintained to support equitable healthy growth among all children [29].

Since its inception, rules outlined in the HHFKA have been challenged, allowing schools implementing the NSLP/SBP to change the meal pattern. Initially, waivers were granted due to supply issues (especially related to wheat). In 2018, a significant policy change issued flexibilities, relaxing nutrition standards for milk, whole grains, and sodium [41,42,43]. These flexibilities allowed for an alternation to the original requirements set forth by the HHFKA. Specifically, the flexibilities allowed schools to offer flavored, low-fat milk (1 percent fat) for students in grades K through 12; serve only 50 percent of the weekly grains in the school lunch and breakfast menus to be whole grain-rich; and allow schools to serve Target 1 sodium levels (≤1420 mg for 9–12th grade, ≤1360 mg for 6–8th grade, and ≤1240 mg for K-5th grade) [41,42,44]. At the time, the administration cited low school meal participation rates and the need to address the concerns of schools (lack of access to product, lower participation rates, etc.) [41,42,43,44]. This rule was overturned in federal courts for lack of specificity, but due to the public health emergency status, it was allowed and remained in effect until July 2022 [43].

The NSLP/SBP are part of a complex system involving food suppliers, school food service directors, and student consumers. Any policy change to the NSLP/SBP impacts diverse actors within each part of this system. The decisions they make in response to a policy change can result in disruptions, inefficient implementation, and ultimately patterns of youth food consumption and nutrition that diverge from the intended goals of the policy. The purpose of this study is to understand the reasons why school food service directors chose to implement the flexibilities and examine the impact of the implementation of the wheat, sodium, and milk flexibilities to the NSLP/SBP on meal participation.

We use the Consolidated Framework of Implementation Research (CFIR), an implementation science framework to measure factors associated with effective implementation [45]. The Consolidated Framework of Implementation Research provides constructs arranged across five domains that can be used for systematically assessing implementation.

## 2. Materials and Methods

This study involved a mixed-method approach to understand how 2018–2022 policy changes (flexibilities) impacted the NSLP/SBP programs and the overall school food system. This study was approved by the Washington University, Human Subjects Research Office (IRB 202009058).

### 2.1. Participants

One hundred and forty-four (144) district food service directors responded to the survey out of 557 invited districts (response rate of 26%). All public school districts in Missouri were invited to take the survey. After incomplete surveys were removed, we were left with 118 responses (completion rate of 82%). In addition, three Missouri school district food service directors representing various school district sizes, locations, and demographics agreed to participate in a semi-structured qualitative interview. All participants were English speaking.

### 2.2. Instrumentation: Quantitative

We first used the CFIR to develop a survey and administer it to Missouri school district food service directors in order to determine whether schools implemented all or some of the flexibilities, which flexibilities were implemented, when flexibilities were implemented, reasons why schools chose to implement a flexibility, and factors related to implementation. Survey questions included multiple choice, binary choice (yes/no), and open text response. Table 1 outlines the survey items as aligned with the CFIR. School district food service directors were recruited through email. Those who agreed to participate completed an electronic (Qualtrics) survey.

### 2.3. Quantitative Data Analysis

Survey responses were analyzed using descriptive techniques, considering proportions of respondents answering a question in a particular way. To determine how implementation of flexibilities affected NSLP/NSB participation, we merged our survey with school-level meal count data from the MO Department of Elementary and Secondary Education (DESE) from the years 2014/15–2019/20 (before and after the new executive order was issued). Using inferential techniques, we conducted ordinary least square (OLS) regression models to examine how flexibility adoption related to the number of meals served:

$$y_{i\in j,t\in m,y}=\beta_{0}+\beta_{1}policy_{i,t}+\beta_{2}\%{enroll}_{i,t}+\beta_{3}{season}_{m}+{County}_{j}+{Year}_{y}+\varepsilon_{i,t}$$

where the served meals’ count (total, paid, and free/reduced) of a school *i* (in county *j*) at time *t* (*y* school year, on *m* month), *y_i__∈__j,t__∈__m,y_*, is a function of policy implementation, *policy_i,t_* controlling the number of enrolled students for lunch/breakfast and monthly seasonality, *season_m,_* as well as county and year fixed effects, *county_j_* and *year_y_*, which capture socioeconomic characteristics at the county level and macro-economy changes, respectively. All analyses were completed using STATA (Statacorp, College Station, TX, USA, version 17, 2021).

Based on our survey sample of districts, our analytic sample includes 528 schools (public, public-charter, public residential child care institutions, and schools for disabled students) from 73 counties in Missouri. In total, 468 of the schools in our sample implemented flexibilities. Each school provides monthly (June and July are excluded due to summer vacation) served meals and enrollment data between 2014–2015 and 2019–2020 school years. The averages of monthly served lunches and breakfasts (total, paid, and free/reduced) across years are represented in Table 2.

### 2.4. Instrumentation: Qualitative

To understand how school districts made implementation decisions and how external agents affect the implementation of flexibilities to the NSLP/SBP standards, we conducted qualitative interviews with school food service directors. We used the CFIR to develop the semi-structured interview guide, which specifically examines characteristics of the flexibilities that resulted in the school district’s decision to adopt flexibilities (i.e., intervention characteristics—relative advantage, evidence strength, cost, and design quality), how well the flexibilities aligned with the school district operations and processes (i.e., inner setting—compatibility, available resources, and leadership engagement), and how the external setting affected implementation of the flexibilities in schools (i.e., outer setting—external policies and agents). The guide was tested for understanding by two field experts: one school-level personnel and one previous employee of USDA.

### 2.5. Qualitative Data Analysis

Key informant interviews were completed in April and May 2021 by three research team members. All interviews lasted between 45 and 60 min and were recorded and professionally transcribed. A codebook was developed utilizing the CFIR domains, and all transcripts were coded by two research team members. Any coding conflicts were discussed between the pair of coders until a consensus was reached. Thematic analysis was completed and based on the CFIR.

## 3. Results

### 3.1. Quantitative Results

When considering student demographics, our survey respondents were fairly representative of the state of Missouri. Concerning race, 87% of the students in surveyed schools identified as white compared to 84% in non-surveyed schools, while 7% of the students in surveyed schools identified as black compared to 10% in non-surveyed schools. Concerning poverty status, 55% of the students in surveyed schools qualified for free and reduced-price lunches compared to 56% in non-surveyed schools; at the school level, 20% of the surveyed schools were Community Eligibility Provision (CEP) eligible compared to 21% of non-surveyed schools.

*Who adopted the policy change, what did they adopt, when did they do it, and why?*

In our Qualtrics survey, 103 out of 118 (87%) respondents adopted “any” of the flexibilities. The most common adoption was wheat flexibilities (82%), followed by milk flexibilities (81%), and sodium flexibilities (51%). Not all adopters implemented the change in each subsequent year; collectively, 43% of adopters implemented the change in 2018, 52% implemented the change in 2019, and 47% implemented the change in 2020. Of these adopters, the most common reason was to serve more food (74%), followed by meeting the preferences of students (72%), having more variety in their menu (62%), responding to vendors (24%), saving money (11%), and logistical convenience (7%). Additionally, when asked how important children’s health was in their decision to adopt the policy on a 4-point Likert scale, 39% reported it was “very” important, and 48% reported it was “extremely” important. Only 12% reported that it was moderately important, and less than 1% reported that it was slightly important. Eighty three percent (83%) of schools that adopted any of these flexibilities reported increased satisfaction from students compared to only 36% of schools that did not adopt these flexibilities. For schools that did adopt these flexibilities, 74% reported serving more food, and 59% reported serving more milk.

*How many more meals were served?*

After merging our district-level survey data from Qualtrics with 2015/16–2019/20 school-level meal count data from the MO Department of Elementary and Secondary Education (given the differences in meal services during the pandemic, we did not merge our survey data with administrative data from DESE for the 2020–2021 school year), we explored the relationship between policy adoption and meals served. Leveraging both policy adoption (yes/no) and time of policy adoption (year), we see (Table 3) that the adoption of flexibilities was associated with an average of 326 (standard error = 35; *p*-value < 0.001) more lunches being served per month and an average of 327 (standard error = 32; *p*-value < 0.001) more breakfasts being served per month. For the 2018/19 school year, this represents roughly an 8.3% increase in the amount of lunches and breakfasts served. However, it is important to note that these treatment effects were heterogeneous. Specifically, monthly paid lunches decreased by 291 (standard error = 22; *p*-value < 0.001), while free/reduced lunches increased by 617 (standard error = 37; *p*-value < 0.001); similarly, paid breakfasts decreased by 59 (standard error = 10; *p*-value < 0.001), while free/reduced breakfasts increased by 386 (standard error = 28; *p*-value < 0.001). These estimates account for lunch and breakfast enrollment and seasonality (month) effects, as well as geographic (country) and temporal (year) fixed effects. Additionally, as some schools terminated adopted flexibilities in subsequent years, we censored these estimates, so that school meal data were removed from the sample for years in which adopted policies were later terminated.

### 3.2. Qualitative Results

We conducted three interviews with school district food service directors. The school district food service directors represented varying levels of flexibility implementation. The themes are organized by the domains within the CFIR.

*Which characteristics of flexibilities resulted in the school food service directors’ decision to implement? (CFIR domain: intervention characteristics).*

#### 3.2.1. Relative Advantage

Relative advantage relates to the stakeholders’ perception of the advantages of implementing the intervention. Student participation and product availability in the NSLP and SBP were cited most by informants in deciding to implement flexibilities.

*Yeah. I like the waivers. Participation has gone up, our goal is to feed kids.*

In addition, informants commented that there was an added convenience when manufacturers were able to provide products that met the new flexibilities, actually allowing them to more easily meet the NSLP guidelines, specifically related to sodium.

*So, the sodium one is mostly because we’re half-convenience and half-speed-scratch cooking, so with the convenience items, clearly they’re already prepared, seasoning’s already been added, it’s more of a heat-and-serve-type situation.*

#### 3.2.2. Cost

The cost construct includes the costs associated with implementing the intervention and can include supply and opportunity costs. All the key informants noted that they made their choices on which products to buy mostly by the cost of the product (and supply and demand). Even for those school food service directors who had decided to implement the flexibilities, they might still look for lower sodium or whole-grain products unless the cost was exorbitant. Cost differences were also noted based on purchasing power between small and large school districts. Small school districts do not receive discounts because they simply are not buying as much product at any given time.

*Because my district is so small, I don’t really have a lot of buying power, so commanding a large discount is not necessarily something that the distributors or vendors are looking for.*

Finally, product supply (intervention design and availability) was noted as a major reason for deciding whether or not to adopt the flexibilities. Milk purchase choice, in particular, was limited in Missouri, requiring many to implement the flexibility due to lack of supply. Fee-for-service items were also not as readily available, driving schools’ decisions.

*Missouri reduced the amount of fee-for-service items. So, that definitely limited a lot of manufacturers in giving us different options.*

*How well did the flexibilities align with school district operations and processes? (CFIR domain: inner setting).*

#### 3.2.3. Compatibility

Compatibility is the degree of fit with values attached to the intervention by involved individuals (i.e., students, school food staff, etc.) and how these align with their norms, values, and needs. Two of the informants, in particular, noted that they did not implement the wheat flexibility because they had already established a food culture of whole grain as the more healthy option.

*Now, the only reason why I didn’t do the wheat one was basically because I feel our food culture here at this district has gotten used to the whole grain, so why change it?*

Other school food service informants noted that even though they wanted to make sure participation remained high, they wanted to be consistent and not keep changing the food environment.

Allowing the sodium flexibility was mostly favored by those interviewed. They felt that the products with extremely low sodium were not palatable and that it would take a few years before students would become accustomed to Target 3 sodium guidelines.

*So, if the flexibilities do not go into play, then it’ll be, I would say, probably several years before palates change again and the kids are getting used to it. When all the new regulations came out, it took a while to trickle down and be part of the school culture- they are used to those types of flavors and satiety factors.*

#### 3.2.4. Available Resources

The available resources domain is the level of resources dedicated for implementation to maintain ongoing operations. All the food service informants commented on the need for process change and challenges related to any policy change affecting the lunch program. They specifically cited that there was not enough labor or time to cook all food from scratch and reported an increased reliance on processed food.

*The other thing is, most schools have gone away from labor, because they have cut labor, so they need the processed products, and processed products has a bad connotation.*

*I would bet 50% of the schools in the country don’t have ovens in their facilities, because they’ve been taken out and they haven’t been replaced.*

#### 3.2.5. Leadership Engagement

Leadership engagement is the commitment or involvement of leaders or managers in implementation. All of the school food service directors felt that they had support from their leaders and full authority to make decisions. School leaders essentially trusted their school food directors to make the right decision for the school.

*Did the external setting affect implementation of the flexibilities in schools? (CFIR domain: outer setting).*

#### 3.2.6. External Policy

The external policies construct includes policies and regulations, external mandates, and guidelines. Regardless of whether or not schools implemented the flexibilities, all informants agreed that it was difficult to keep up with the ever-changing policies.

*Some of the conversations were going and scale back to the point of I’m just going to stick back to what we were doing before and wait this thing out. We have to weigh a lot of things before we as school food authorities just simply run with the latest piece of paper that’s come down the pipe.*

## 4. Discussion

This mixed-method study is the first to examine and document school district decisions in implementing the 2018 policy to allow wheat, sodium, and milk flexibilities across a state. Both our qualitative and quantitative results indicate that a majority of schools decided to implement the flexibilities. The most common reasons cited for deciding to adopt a flexibility was to increase participation and meet the preferences of students. Key informants also noted that the availability of products was important in their decision-making, especially as the pandemic forced school districts to adapt their processes and increased demand for certain products.

Many school districts self-reported that adoption of flexibilities resulted in a significant increase in their overall NSLP/SBP participation. Further analysis of administrative data confirmed this assertion. When examining NSLP/SBP participation, we see that the adoption of flexibilities was associated with more lunches and breakfasts being served per month. This increase was noted specifically among free and reduced-price lunches and breakfasts. These heterogeneous effects point to an important observation—students with the means to pay full-price for lunch chose other options when less healthy menus (i.e., those that included more sodium, more fat, and less whole grain) emerged, while free and reduced-price meal participation increased.

In addition, our results indicate a notable misalignment between a school district’s decision to adopt flexibilities and offer less healthy options and concern for student health. Over 85% of school district food directors reported concern for student health as very important in driving their decision to implement flexibilities. The same number decided to implement some form of flexibility, decreasing the overall nutritional value of the meals served.

While studies have shown that the benefits of providing food to low socio-economic status (SES) students as part of the NSLP/SBP can outweigh the costs, it is unknown how offering meals that do not fully meet dietary guidelines might result in inequities in health outcomes for low SES students. Several studies have documented the potential for negative health impacts from the implementation of sodium, wheat, and milk flexibilities. Jackson et al. [46] note that even with the successful implementation of sodium Target 1 (1230 mg–1420 mg) reductions, nine out of ten children still consume too much sodium [46]. Therefore, further delaying the implementation of sodium Target 2 (935 mg–1080 mg) and Target 3 (640 mg–740 mg) will further result in consumption of too much sodium, increasing their risk of high blood pressure, heart disease, and stroke [46,47]. In addition, studies have shown that eating more whole grains is associated with a reduced risk of heart disease, stroke, and diabetes, yet children, on average, consume too few whole grains and too many refined grains [48,49,50,51,52,53,54]. While the impetus for issuing the whole grain flexibility was related to lack of supply, the “USDA conceded in the final rule that 85 percent of schools have not requested waivers and are providing children with appealing whole-grain options” [44]. Finally, the original HHFKA standards that permitted only plain or flavored fat-free milk and plain low-fat milk were developed because the National Academy of Medicine noted that offering sugar-added flavored low-fat milk would push school meals past overall calorie limits [27]. The 2015 Dietary Guidelines also recommended “reducing the intake of added sugars” such as those in chocolate- and strawberry-flavored milk [28].

Findings from this study can inform the design and implementation of future policies related to the NSLP/SBP. First, it appears that for many students, especially those that on low-incomes, meals served under the new flexibilities were more appealing. Given the prevalence of child food insecurity, when deciding school food policies, stakeholders should consider the trade-offs between ensuring that more students eat school meals and ensuring that students eat healthy school meals. As both sentiments are essential for student health, ultimately, stakeholders should consider how to serve healthy and nutritious foods that appeal to the tastes of all students. When designing school meal policies, stakeholders should also consider how intervention characteristics, as well as aspects across “inner” and “outer” settings, will relate to the implementation of policies. For example, food cost and product availability should be important considerations when designing and implementing school meal policies. Finally, when considering the ever-changing policy landscape around school meals, policy-makers and other stakeholders should consider ways to ensure that schools have adequate time and resources to change.

### Limitations

There are three main limitations in this study. First, although our survey respondents were fairly representative of the state, it is possible that certain unobserved characteristics caused some districts to respond to the survey, which may bias our results. In addition, our qualitative results only represent a few school districts throughout the state. Although we chose these school districts based on their implementation status and difference in size, student population, and location, our qualitative results may not be generalizable. Second, we are not able to observe the exact foods or nutritional quality of the foods served or consumed before and after the policy change, which limits our ability to understand what is driving the uptake in meals after policy adoption, as the health consequences of this increase. Finally, as we cannot observe other measures related to children’s health, it is difficult to demonstrate the trade-offs between serving more meals and serving fewer, healthier meals. Future studies should seek to understand how this policy and meals served relates to children’s health and hunger status.

In February 2022, the USDA issued a final rule to extend the flexibility waivers for milk, sodium, and wheat. The new standards will take effect on 1 July 2022 and apply to the 2022–2023 and 2023–2024 school years. The changes are a bit different from the 2018 flexibilities but are still a departure from the original HHFKA requirements [55]. For instance, instead of 50 percent of required whole grain, they are requiring 80 percent. In addition, the milk requirement allows for flavored, low fat milk but at a cost to the student as a competitive beverage [55]. Despite allowing the continued use of waivers, there is an increased focus by the Biden administration on nutrition: they recently announced a National Conference on Hunger, Nutrition, and Health tasked to improve food access and affordability, integrate nutrition and health, and empower all to have access to healthy choices (including school policies).

The USDA will issue a proposed rule for future nutritional standards for stakeholder comment this fall. The final rule will be issued in time to prepare for the 2024–2025 school year. Changes could result in inequities among food served to students in vulnerable schools, specifically those in rural and low SES areas who do not have the same product availability and access. Given our findings, continued research is needed to determine how the availability and increased uptake of school meals that do not fully meet dietary guidelines by low-income students result in inequities in health outcomes, as well as how these results may differ across geographic (e.g., urban and rural) contexts. Research should inform final standards to ensure equitable access and uptake of nutritious meals.

## Figures and Tables

**Table 1:** Description of Consolidated Framework of Implementation Research constructs used in survey and interviews.

CFIR Domain and Construct	Survey and Interview Questions
Intervention characteristics: characteristics of the flexibilities that resulted in a school district’s decision to adopt flexibilities Relative advantage Evidence strength Cost Design and package quality	In 2018, the federal government relaxed some of the regulations on school meals. Did you implement one of these flexibilities? Why did you implement these changes? Have you noticed a shift in overall milk consumption as a result of the changes? Have you noticed a shift in meal consumption as a result of the changes? To what degree did the evidence influence your opinion of the current and proposed NSLP guidelines?
Inner setting: how well the flexibilities aligned with the school district operations and processes Compatibility Available resources, leadership engagement	What changes have you made as a result of the flexibilities? How important was children’s nutrition in the decision to implement or not implement changes? Do you think student satisfaction has changed? What type of support does leadership in your school provide in terms of implementing the flexibilities?
Outer setting: how the external setting affected implementation of the flexibilities in schools External policies and agents	Do you use an external food vendor? What type of support does the USDA offer in terms of understanding and implementing the flexibilities? Have you been able to secure enough products from your suppliers to implement the new flexibilities?

**Table 2:** Average number of served lunches and breakfasts per month.

School Year	2014–2015	2015–2016	2016–2017	2017–2018	2018–2019 *	2019–2020 *
Lunch						
Total	4643.1	4584.7	4118.3	4062.2	3940.5	3645.6
Paid	1795.7	1764.2	1606.1	1629.7	1606.5	1511.3
Free/reduced	2847.4	2820.5	2512.1	2432.5	2334.0	2134.2
Breakfast						
Total	2018.5	2030.1	1878.2	1884.7	1884.4	1807.5
Paid	382.4	400.1	392.1	424.3	442.5	436.3
Free/reduced	1636.1	1630.0	1486.2	1460.5	1441.9	1371.2
District Enrollment	7084.5	7030.7	6509.4	6505.0	6529.0	6416.0

* Designates years that nutrition standards were relaxed.

**Table 3:** Policy impacts on served meals.

	Lunch			Breakfast		
Items Studied	Total	Paid	Free/Reduced	Total	Paid	Free/Reduced
	(1)	(2)	(3)	(4)	(5)	(6)
Policy	326.0 ***	−291.1 ***	617.0 ***	326.9 ***	−59.3 ***	386.2 ***
	(35.1)	(22.2)	(37.3)	(32.2)	(9.9)	(28.2)
Seasonality (month)	Y	Y	Y	Y	Y	Y
Enrolled student	Y	Y	Y	Y	Y	Y
Observations	29164	29164	29164	29164	29164	29164
Adjusted *R*^2^	0.688	0.630	0.421	0.337	0.261	0.346
AIC	510,367.6	483,618.6	513,788.1	505,338.5	436,674.1	497,575.8
BIC	511,104.6	484,355.5	514,525.0	506,075.5	437,411.1	498,312.8

Notes: Standard errors in parentheses; seasonality (monthly) and the numbers of students enrolled for lunch/breakfast (monthly) controlled; county and school year fixed effects controlled; estimates are right censored, such that schools that implemented the policy in 2018–2019 but not in 2019–2020 were not included in the latter estimates; AIC: Akaike information criterion; BIC: Bayesian information criterion; *** *p* < 0.001.

## Data Availability

Data will be made available upon request.
